# Genetic Changes of Reemerged Influenza A(H7N9) Viruses, China

**DOI:** 10.3201/eid2009.140250

**Published:** 2014-09

**Authors:** Jing Lu, Jie Wu, Dawei Guan, Lina Yi, Xianqiao Zeng, Lirong Zou, Lijun Liang, Hanzhong Ni, Xin Zhang, Jinyan Lin, Changwen Ke

**Affiliations:** Guangdong Provincial Center for Disease Control and Prevention, Guangzhou, China

**Keywords:** Avian influenza, H7N9, H9N2, reassortment, Guangdong, China, viruses, influenza

**To the Editor:** From March 30, 2013, through April 8, 2014, a total of 401 human infections with novel avian influenza A (H7N9) virus were reported in China (*1*). In the initial wave from February through May 2013, cases were laboratory confirmed for 133 patients (45 died), mainly in eastern China. From June through early October 2013, only 2 laboratory-confirmed cases were reported in China. One of these, identified on August 10, 2013, was the first case of influenza A(H7N9) virus infection in Guangdong Province (strain A/Guangdong/HZ-01/2013). However, a second wave of influenza A(H7N9) virus infection began on October 14, 2013 (*2*). As of April 8, 2014, a total of 266 laboratory-confirmed cases had been reported, mainly in Zhejiang Province in eastern China (92 cases, 37 deaths) and Guangdong Province in southern China (99 cases, 30 deaths).

Previous sequencing studies suggested that 6 of the 8 influenza A(H7N9) virus RNA segments were acquired from influenza A(H9N2) virus. This acquisition process involved at least 2 steps of sequential reassortment; the most recent event most likely occurred in the Yangtze River Delta area of eastern China (*3*–*5*). To date, nearly all analyses have been performed by using sequences obtained from viruses isolated during the first wave of infection; changes associated with viruses isolated during the second wave are largely unknown (*6*). We therefore conducted phylogenetic analyses of whole-genome sequence data for 15 influenza A(H7N9) viruses isolated from human patients in Guangdong from November 4, 2013, through January 15, 2014. 

We estimated maximum-likelihood trees for all 8 RNA segments by using MEGA version 5.2 and the general time-reversible model (*7*). RNA segments encoding the hemagglutinin, neuraminidase, and matrix genes of A/Guangdong/H7N9 viruses isolated after November 2013 were genetically similar to those of A/Guangdong/HZ-01/2013 and H7N9 strains from the first wave of influenza (Technical Appendix). An additional 4 RNA segments (nonstructural protein [NS], nucleocapsid protein [NP], polymerase basic proteins [PB] 1 and 2) of A/Guangdong/H7N9 influenza viruses isolated after November 2013 were clustered with A/Guangdong/HZ-01/2013 virus and were divergent from all currently sequenced subtype H7N9 viruses from the first wave in eastern China. The only exception was the NP segment of A/Guangdong/SZ-026/2014, which was found segregated into a separate cluster with subtype H9N2 viruses from Shandong Province. Moreover, analyses showed that RNA segments encoding NS, NP, PB1, and PB2 of A/Guangdong/H7N9 isolated after November 2013 were most similar to the same segments from influenza A(H9N2) viruses that had recently circulated in Guangdong (Technical Appendix Figure, panels D–G). That is, NS, NP, PB1, and PB2 showed greater similarity to local subtype H9N2 viruses from Guangdong than to subtype H7N9 viruses from the first wave of influenza.

Notably, 2 separate clusters were observed for the phylogenetic tree of the RNA segment encoding the polymerase acidic gene (Technical Appendix Figure, panel H). A/Guangdong/HZ-01/2013-like viruses clustered with subtype H7N9 viruses from the first wave of influenza. However, A/Guangdong/DG-02/2013-like viruses were clustered with subtype H9N2 influenza viruses circulating in Guangdong, suggesting that recent reassortment with circulating subtype H9N2 viruses occurred after the first case of infection with influenza A(H7N9) virus reported in Guangdong (Technical Appendix Figure, panel H).

This study provides evidence that influenza A(H7N9) viruses isolated during the second wave of influenza in Guangdong differ genetically (in 5 of the 8 RNA segments) from that of influenza A(H7N9) viruses isolated during the first wave. High similarity of these 5 segments with those of locally circulating subtype H9N2 viruses suggests that rapid and continued reassortment with circulating subtype H9N2 viruses occurred during the second wave of the influenza A(H7N9) virus epidemic. Because reassortment and genetic changes can contribute to host fitness and infection capacity of reemerged influenza A(H7N9) viruses, studies of pathogenicity and transmission, to reveal the exact role of each genetic alteration, are needed.

Technical Appendix Phylogenetic tree of the 8 RNA segments from influenza A(H7N9) virus isolates.
