# Supplementary material for: Genetic Changes of Reemerged Influenza A(H7N9) Viruses, China
Source: Emerg Infect Dis. 2014 Sep;20(9):1582–3. doi: 10.3201/eid2009.140250 (PMC4178397; doi:10.3201/eid2009.140250)
Supplement: Technical Appendix — Phylogenetic tree of the 8 RNA segments from influenza A(H7N9) virus isolates. [file 14-0250-Techapp-s1.pdf]

Article DOI: http://dx.doi.org/10.3201/eid2009.140250

Genetic Changes of Reemerged Influenza A(H7N9) Viruses, China

Technical Appendix

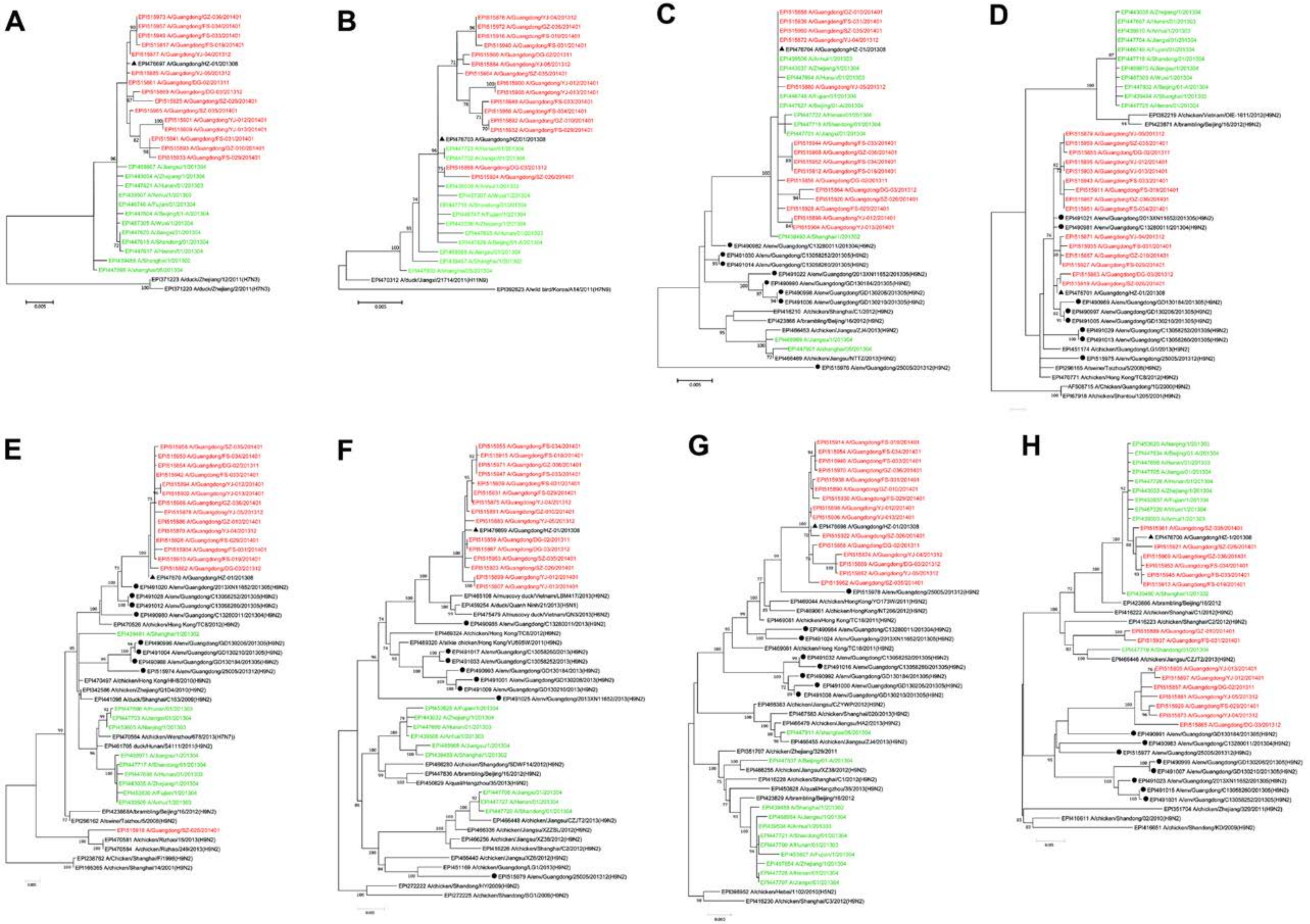

Technical Appendix Figure. Phylogenetic tree of the 8 RNA segments from influenza A(H7N9) virus isolates. Nucleotide sequences were analyzed by using the maximum-likelihood method (A–G). Supporting bootstrap values >70 are shown. Scale bars indicate nucleotide substitutions per site. The collection times (year, month) and cities for influenza A(H7N9) virus Guangdong strains are indicated. Green, influenza A(H7N9) viruses isolated during the first influenza wave; red, influenza A(H7N9) virus isolated during the second wave; triangles, A/Guangdong/HZ-01/2013 isolated between the first and second waves on July 28, 2013; circles, influenza A(H9N2) viruses circulating in Guangdong; GZ, Guangzhou city; DG, Dongguan city; YJ, Yangjiang city; FS, Foshan city; SZ, Shenzhen city. A) hemagglutinin, B) neuraminidase, C) matrix protein, D) nonstructural protein, E) nucleocapsid protein, F) polymerase basic protein 1, G) polymerase basic protein 2; H) polymerase acidic protein.
